# Analysis of Hyperphagia Questionnaire for Clinical Trials (HQ-CT) scores in typically developing individuals and those with Prader-Willi syndrome

**DOI:** 10.1038/s41598-023-48024-5

**Published:** 2023-11-23

**Authors:** Lisa Matesevac, Caroline J. Vrana-Diaz, Jessica E. Bohonowych, Lauren Schwartz, Theresa V. Strong

**Affiliations:** 1 Foundation for Prader-Willi Research, Covina, CA USA; 2 Department of Rehabilitation Medicine, University of Washington, Seattle, WA USA

**Keywords:** Clinical genetics, Genetics, Medical research

## Abstract

The Hyperphagia Questionnaire for Clinical Trials (HQ-CT) is an observer-reported outcome measure that has been widely used in interventional studies to assess changes in hyperphagic behaviors in individuals with Prader-Willi syndrome (PWS). However, HQ-CT scores in the wider PWS population and the general population have not been reported. Here we report HQ-CT scores from more than 400 individuals with PWS and 600 typical individuals, aged 5–26. Overall, HQ-CT scores were significantly higher in those with PWS compared to typically developing individuals at all ages evaluated. In addition, while HQ-CT scores in the typically developing population decreased with age, scores increased with age in PWS. To further understand the variability of HQ-CT scores in the PWS population, semi-structured interviews were conducted with caregivers of a small subset of adults with PWS who had unexpectedly low HQ-CT scores. These caregivers reported that strict adherence to a food routine, food security measures and supervised food preparation reduced the frequency and intensity of hyperphagic behaviors measured by HQ-CT. Thus, hyperphagic behaviors are captured by the HQ-CT for most individuals with PWS, but for some individuals residing in settings with highly structured food routines, HQ-CT scores may not fully reflect the extent of PWS-associated hyperphagia.

## Introduction

Prader-Willi syndrome (PWS) is a rare neurodevelopmental disorder affecting approximately 1 in 10,000 to 30,000 individuals^1–3^. PWS results from loss of expression of the paternally expressed, imprinted genes on chromosome 15q11.2-q13, due to a paternal deletion (~ 60% of individuals), maternal uniparental disomy (UPD, ~ 35% of individuals), or an imprinting defect (ID, 3–5%). Common characteristics of the syndrome include hypotonia and failure to thrive in infancy followed by the onset of hyperphagia during childhood. Hyperphagia leads to morbid obesity if food intake is not strictly controlled. Other features of PWS include multiple endocrinopathies, altered body composition, cognitive impairment, sleep disturbances, scoliosis, hypogonadism, and a challenging neurobehavioral phenotype^3,4^.

The hallmark symptom of PWS is hyperphagia, with an onset of early to mid-childhood. Miller and colleagues described seven nutritional phases of PWS, beginning with in utero growth restriction (Phase 0) followed by difficulty feeding in infancy (Phase 1a), changing to steady growth at a typical rate in toddlerhood (Phase 1b), weight gain without changes in caloric intake and increased interest in food in early childhood (Phase 2a and 2b), and hyperphagia onset at a median age of 8 years old (Phase 3)^5^. Hyperphagia in PWS is characterized as an intense persistent sensation of hunger accompanied by food preoccupations, an extreme drive to consume food, food-related behavior problems, and a lack of normal satiety^6^. This characteristic of PWS significantly impacts all aspects of life for the person with PWS and their family, substantially limiting independence. Management of PWS-associated hyperphagic behaviors typically falls to a family member, and a high level of caregiver burden is associated with the disorder^7^, with higher levels of hyperphagia correlated with increased caregiver burden^8^. Hyperphagia typically persists through adulthood, but in rare cases, adults with PWS have been reported to enter “Phase 4”, where food seeking behaviors decrease^5^. It remains unclear, however, if “Phase 4” represents a true diminution of hyperphagia or rather reflects a decrease in observable hyperphagic behaviors due to long-term exposure to strict and consistent food management^9^.

Over the last decade, a number of drugs aimed at reducing hyperphagia in PWS have been evaluated in clinical trials (e.g., ClinicalTrials.gov Identifiers NCT01279151, NCT03649477, NCT03714373, NCT03274856, NCT05322096, NCT05153434). The primary endpoint for measuring hyperphagia in these trials is the Hyperphagia Questionnaire for Clinical Trials (HQ-CT)^10^, a nine-question, observer-reported clinical outcome assessment adapted for clinical trials from the PWS-specific Hyperphagia Questionnaire by Dykens and colleagues^11^. This instrument is completed by a parent/primary caregiver of the person with PWS and assesses the frequency and intensity of hyperphagic behaviors over the past two weeks. Among the items assessed by this instrument is the frequency of food seeking activities, the intensity of outbursts related to being denied a desired food, and the persistence in talking about and asking or looking for food. Each item is scored from 0 to 4, for a total possible score of 36.

Although the HQ-CT has been widely used in the research and clinical communities and has been accepted by regulatory agencies as a measure of observable hyperphagic behaviors in PWS, no previous study has examined normative data on this questionnaire. Here, we examined HQ-CT scores in a large population of individuals with PWS compared to typically developing children and young adults. To further understand the variability of HQ-CT scores in the PWS population, and in particular, the factors associated with low scores in some adults with PWS, we used a semi-structured interview format for caregivers of a small subset of adults with PWS who had consistently low scores on the HQ-CT.

## Methods

The HQ-CT was administered electronically to caregivers of individuals with PWS who were enrolled in the “PATH for PWS” prospective natural history study (NCT03718416) in the Global PWS Registry^12^. Additional information including demographic information, height and weight was provided by the respondent. Participants (the person with PWS) were classified as underweight, normal weight, overweight or obese based on calculated body mass index (BMI) for age. For participants under the age of 20, BMI under the 5th percentile is considered underweight, BMI of 5th to 85th percentile is considered normal weight, BMI above the 85th percentile is overweight, and above the 95th percentile is obese. For participants aged 20 or above, BMI of under 18.5 was considered underweight, BMI of 18.5–24.9 was considered normal weight, a BMI of 25–30 was considered overweight, while BMI of > 30 was obese.

Caregivers of typically developing individuals 5–26 years of age completed the HQ-CT via SurveyMonkey. The instructions, questions and format of the HQ-CT matched that found in the Global PWS Registry. North Star Institutional Review Board (IRB) reviewed the study protocol and questionnaire for data collection in the typically developing population and provided an exemption, as all information collected was de-identified (NB100032). Recruitment for the study was conducted via a social media campaign in PWS specific groups, where caregivers were encouraged to complete the survey for their typical children and share the survey with family and friends who did not have children with PWS. Assent was requested if the person for whom the survey questions were being completed was age 12 or older. In addition to the HQ-CT questions, respondents reported the age of the person for whom the survey was being completed, whether the person lived in a home with someone with PWS, if the person was related to an individual with PWS, the weight category of the person for whom the survey was completed, as well as whether the person had a neurodevelopmental disorder or if they were on a medication that might impact appetite. Respondents were able to complete the survey for up to 4 typically developing individuals in their care.

Analysis of demographic information includes proportions and means with standard deviation. Two-sample independent *t* tests and analysis of variance (ANOVA) tests (with Tukey HSD post-hoc tests) were used for bivariate analysis, and a *p* value of < 0.05 was considered for statistical significance.

For the semi-structured interviews regarding food behaviors of adults with PWS reported to have low HQ-CT scores, caregivers of participants in the “PATH for PWS” natural history study were invited to participate in an interview if the following inclusion criteria for consideration were met: the participant must not have been enrolled in a clinical trial or have had any recent major life changes (e.g., death in the family, change in living environment, etc.) and the caregiver must have reported that they were “very familiar” with the participant’s day-to-day activities. Of the 459 individuals who met this criteria, 55 reported HQ-CT scores of 7 or lower in their loved one with PWS aged 18 or older. Of these, 32 participants were identified whose scores on the most recently completed HQ-CT remained at 7 or lower, and who met the inclusion criteria. The 32 respondents were contacted via email and text and provided details of the study. Seventeen respondents expressed an interest in participation, consented and completed a telephone interview exploring their experiences with their loved one with PWS regarding food and non-food related behaviors, daily activities in and away from the home and how the family manages the food needs of the person with PWS. North Star IRB reviewed the protocol, consent and interview questions and exemption was provided (NB100031). All interviews were conducted by the same person (LM) using a standard format and set of questions, with interview times ranging from 1–2 h per respondent. Interviews were recorded on a password protected device and used to transcribe de-identified information and obtain relevant quotes from the respondents.

### Ethics approval and consent to participate

The PATH for PWS study was conducted in accordance with the Declaration of Helsinki and approved by the Institutional Review Board of New England IRB (Global PWS Registry Protocol #2017–16, D-Dimer sub-study Protocol #2018–59). The Food Behavior Survey for Typically Developing Individuals was conducted in accordance with the Declaration of Helsinki and was reviewed and exempted by the North Star Institutional Review Board (NB100032) under 45 CRF 46.104(d)(2). The Variability in HQ-CT Scores in Adults with PWS was conducted in accordance with the Declaration of Helsinki and was reviewed and exempted by the North Star Institutional Review (NB100031) under 45 CRF 46.104(d)(2). All legal guardians/legally authorized representatives of the person with PWS completed an informed consent. All legal guardians/legally authorized representatives of the typically developing individuals completed an assent.

## Results

### HQ-CT scores for participants with PWS compared to typically developing individuals

Data on HQ-CT scores in the PWS population were obtained from participants in the “PATH for PWS” natural history study (NCT03718416), a registry-based prospective study of individuals with PWS, age 5 and above, with data entered by the parent or primary caregiver of the person with PWS (the respondent). At enrollment into “PATH for PWS”, the HQ-CT was completed for 646 individuals with PWS. Of those, participants were excluded from this analysis if they were receiving an experimental drug as part of a clinical trial, if the respondent indicated that the participant had experienced a major life change (e.g., change in living condition or health status) in the past 6 months, or if the respondent was not “very familiar" with the participant's daily activity. After excluding participants for these reasons, the HQ-CT scores for 459 individuals with PWS were analyzed. The demographics of the participants is shown in Table 1. The genetic subtype of PWS participants were 55% deletion, 32% uniparental disomy, 2% imprinting defect, 1% other (e.g., translocation, atypical deletion), and 10% don't know/not reported.

**Table 1:** Demographics of PWS and typically developing participants.

Sociodemographics	PWS Participants (N = 459) (number, %)	Typical Participants (N = 614) (number, %)
Age category		
5–11	167 (36.38%)	473 (77.0%)
12–17	129 (28.10%)	112 (18.34%)
18 +	163 (35.51%)	29 (4.72%)
Race/Ethnicity		
American Indian or Alaska native	1 (0.22%)	1 (0.16%)
Asian	13 (2.83%)	5 (8.1%)
Black or African American	8 (1.74%)	3 (4.9%)
Hispanic or Latino	29 (6.32%)	8 (1.3%)
Multi-ethnic	25 (5.45%)	40 (6.5%)
Native Hawaiian or other Pacific Islander	2 (0.44%)	1 (0.16%)
Other	4 (0.87%)	1 (0.16%)
Prefer not to answer	1 (0.22%)	6 (9.8%)
White	376 (81.95%)	541 (88.1%)
Not reported	1 (0.22%)	8 (1.3%)
Current weight category^a^		
Underweight	4 (0.87%)	40 (6.5%)
Normal weight	146 (31.81%)	516 (84.0%)
Overweight	98 (21.35%)	53 (8.6%)
Obese	180 (39.22%)	4 (0.65%)
Don’t know/Not reported	31 (6.75%)	1 (0.16%)
PWS Living Status		
Living with someone with PWS	N/A	161 (26.2%)
Not living with someone with PWS	N/A	453 (73.8%)
Relationship status to someone with PWS		
Sibling	N/A	162 (26.4%)
Cousin	N/A	31 (5.1%)
Other	N/A	12 (2.0%)
Not related	N/A	409 (66.6%)

^a^For those with PWS, weight category was determined by BMI calculated from reported weight and height for each individual. For typically developing individuals, weight status was reported by the respondent, with options of “underweight”, “normal weight”, “somewhat overweight” or “obese”.

For typically developing individuals, 605 respondents (parent/caregiver) reported on 734 individuals in an electronic survey that included the HQ-CT questionnaire. Responses were excluded if the participant had any neurodevelopmental disorder, if they had a medical condition or took a medication that would impact appetite, if they were not in the daily care of the respondent, or if the individual was under the age of 5 or over the age of 26. Thus, HQ-CT scores for 614 typically developing children and young adults were included in this analysis. Table 1 shows the demographics of these individuals. Respondents were also asked to indicate whether the individual they were reporting on lived with someone with PWS. Most of the typically developing participants did not live with someone with PWS (73.8%) and were not related to someone with PWS (66.6%).

**Figure 1:**
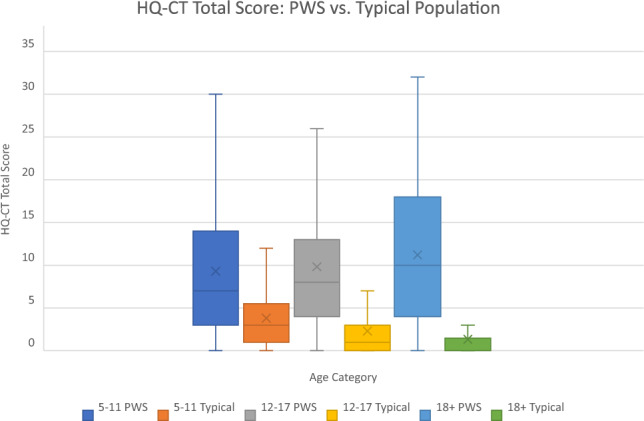
Mean HQ-CT total score: PWS versus typical individuals within age categories. Box and whisker plots of HQ-CT scores in each age category is shown. The middle line of each box is the median, the x is the mean. The bottom and top lines of the box are the 1st quartile and 3rd quartile, respectively, and the whiskers extend to the minimum and maximum values. Ns are as follows: 5–11 PWS: 167, 5–11 Typical: 473; 12–17 PWS: 129, 12–17 Typical: 112; 18 + PWS: 163, 18 + Typical: 29.

The HQ-CT scores by age category (5–11, 12–17, 18 +) of individuals with PWS compared to typically developing individuals is shown in Fig. 1. Among the 459 participants with PWS, the overall mean HQ-CT score was 10.13 (SD: 7.94), and the total scores ranged from 0 to 36. Among 614 typically developing participants, the overall mean HQ-CT score was 3.43 (SD: 3.50), and the total scores ranged from 0 to 22. The mean HQ-CT score was significantly higher among the participants with PWS compared to the typically developing participants (*t* = 18.63, *p* < 0.0001). An increasing linear trend in mean HQ-CT score was found as compared to age in persons with PWS, while scores decreased with age in typically developing children and young adults. For each age category, PWS individuals had a statistically significantly higher mean HQ-CT total score compared to typical individuals (5–11-year-olds: 9.31 vs. 3.82, *t* = 12.35, p < 0.0001, 12–17-year-olds: 9.82 vs. 2.31, *t* = 9.63, *p* < 0.0001, 18 + year olds: 11.21 vs. 1.34, *t* = 6.30, *p* < 0.0001, respectively).

Table 2 shows the bivariate analysis of sociodemographic characteristics and mean HQ-CT score among the individuals with PWS. There was no statistically significant difference in mean HQ-CT total score by age category or by PWS genetic subtype. There was a statistically significant difference in mean HQ-CT total score by current weight category (underweight: 8, normal weight: 8.74, overweight: 8.42, obese: 12.15, F = 7.5, *p* < 0.00001). In assessing the post-hoc tests, those in the obese category had a higher mean HQ-CT total score than both those with normal weight (*p* = 0.0004), and those in the overweight category (*p* = 0.0007).

**Table 2:** Bivariate analysis of sociodemographics and mean HQ-CT total score among individuals with PWS.

Characteristic, N (%)	HQ-CT total score (mean ± SD)
Age category	
5–11, 167 (%)	9.3 ± 7.63
12–17, 129 (%)	9.82 ± 7.67
18 + , 163 (%)	11.21 ± 8.37
Current weight**	
Underweight, 4 (0.87%)	8 ± 16
Normal weight, 146 (31.8%)	8.74 ± 6.93
Overweight, 98 (21.4%)	8.42 ± 6.77
Obese, 180 (39.2%)	12.15 ± 8.43
Missing: 31 (0.16%)	
Genetic subtype	
Deletion, 257 (56.0%)	10.02 ± 8.12
Uniparental disomy, 143 (31.2%)	9.38 ± 7.29
Imprinting defect, 11 (2.4%)	9.82 ± 6.71
Translocation, 4 (0.87%)	13.25 ± 9.64
Other, 3 (0.65%)	20.3 ± 5.03
Don’t know, 41 (8.93%)	12.49 ± 8.66

**p < 0.0001.

Bivariate analysis was performed using ANOVA tests with Tukey HST post-hoc tests.

Table 3 shows the bivariate analysis of sociodemographic characteristics and mean HQ-CT score among the typically developing participants. There was a statistically significant difference in mean HQ-CT total score by age category (5–11-year-olds: 3.82, 12–17-year-olds: 2.31, 18 + year olds: 1.34, F = 14.45, *p* < 0.0001). In assessing the post-hoc tests, those in the 5–11-year-old group had a higher mean HQ-CT total score than both those in the 12–17-year-old group (*p* = 0.0001), and those in the 18 + year old group (*p* = 0.0005). There was also a statistically significant difference in mean HQ-CT total score by current weight category (underweight: 3.5, normal weight: 3.16, somewhat overweight: 5.77, obese: 7.5, F = 11.28, *p* < 0.00001). In assessing the post-hoc tests, those in the ‘somewhat overweight’ category had a higher mean HQ-CT total score than both those with normal weight (*p* < 0.0001), and those in the underweight category (*p* = 0.0085). There was no statistically significant difference in mean HQ-CT total score by PWS living status or PWS relationship status.

**Table 3:** Bivariate Analysis of Sociodemographics and Mean HQ-CT total score among typically developing participants.

Characteristic, N (%)	HQ-CT total score (Mean ± SD)
Age category**	
5–11, 473 (77%)	3.82 ± 3.52
12–17, 112 (18.3%)	2.31 ± 3.26
18 + , 29 (4.72%)	1.34 ± 2.26
Current weight**	
Underweight, 40 (6.5%)	3.5 ± 4.33
Normal weight, 516 (84%)	3.16 ± 3.19
Somewhat overweight, 53 (8.6%)	5.77 ± 4.30
Obese, 4 (0.65%)	7.5 ± 7.59
Don’t know, 1 (0.16%)	
PWS living status	
Living with someone with PWS, 161 (26.2%)	3.09 ± 3.69
Not living with someone with PWS, 453 (73.8%)	3.55 ± 3.42
Relationship Status to someone with PWS	
Sibling, 162 (26.4%)	3.09 ± 3.67
Cousin, 31 (5.1%)	2.87 ± 3.21
Other, 12 (2%)	3 ± 2.13
Not Related, 409 (66.6%)	3.62 ± 3.47

**p < 0.0001.

Bivariate analysis was performed using two-sample t-tests and ANOVA tests with Tukey HST post-hoc tests.

We compared the scores of the 9 individual HQ-CT questions in the typically developing individuals to those with PWS. Individuals with PWS had higher mean individual HQ-CT question scores for all 9 questions compared to typical individuals (all *p* < 0.001, see Supplemental Table 1, Additional File 1). Supplemental Table 2, Additional File 1 shows the bivariate analysis of the individual HQ-CT questions by age categories, PWS living status, and PWS relationship status among the typically developing individuals.

### Caregiver interviews for low scoring individuals with PWS

Although both the means and ranges of HQ-CT scores in individuals with PWS were significantly higher than typical individuals and were higher with increasing age, there was a wide range of HQ-CT scores in individuals with PWS. We were interested in a small subset of individuals with PWS who consistently had low HQ-CT scores, even well after the expected onset of hyperphagia^5^. To better understand the basis of low HQ-CT scores in some adults with PWS, we invited the caregivers of 32 adult PWS participants with HQ-CT scores below 7 at baseline and at their most recent evaluation to participate in a semi-structured interview aimed at understanding the food seeking behaviors of the individual, as well as the food environment. Caregivers of 17 adults with PWS ranging in age from 19 to 61 years of age at the time of enrollment in the PATH for PWS study agreed to participate in the semi-structured interview. Mean HQ-CT scores of these individuals was 3.23 (SD 2.46) at enrollment and 2.35 (SD 1.93) at the most recent data collection point, and 65% of the individuals were overweight or obese. Demographic characteristics of these participants are shown in Table 4 and are consistent with the demographics of the overall PWS sample.

**Table 4:** Demographics of PWS Participants represented in Caregiver Interviews (n = 17).

	Participants (N, %)
Mean age of PWS participant	31.12 ± 11.95
Age range	19–61
Genetic subtype	
Deletion	8 (47.1%)
UPD	4 (23.5%)
Don’t know	5 (29.4%)
Current weight category	
Underweight	0 (0%)
Normal weight	6 (35.3%)
Somewhat overweight	3 (17.7%)
Obese	8 (47.1%)

Caregivers were asked about food seeking behaviors in the person with PWS, and strategies, if any, the caregiver employed to manage food intake and food-related behaviors of these individuals. A selection of responses is presented in Table 5. All respondents reported actively managing the food intake of the person with PWS; 100% of the caregivers reported that they prepare the meals or directly assist the person with PWS in meal preparation to ensure appropriate portion control and meal choices. Repetitive snacks and meal choices (driven by the person with PWS) were reported by 41.2% (n = 7) of the caregivers. The majority, 76.5% (n = 13), reported that the individual with PWS had little to no independence with respect to food choices at home or outside of the home. Consistent with the food behaviors of many individuals with PWS, 64.7% (n = 11) of the respondents report their loved one with PWS asked questions about food throughout the day, including the type, timing, and/or portion of food to be served. A common strategy employed by these caregivers was strict routine and structure regarding food, which included strictly scheduled mealtimes and snack times. Disruption to routine was reported by 88.2% (n = 15) of the respondents as almost never happening, further illustrating how caregivers use this strategy for food behavior management. Situations that deviated from routine were reported to cause significant behavioral difficulties in the person with PWS and were therefore avoided.

**Table 5:** Food control strategies and selected quotes from interviews of caregivers reporting low HQ-CT scores.

Question	Caregiver response
What happens when there is a disruption to food routine?	Increased food questions 47% Negative behaviors 35% Change to routine almost never happens 88%
Typical day regarding meal preparation and food management	Repetitive food/snack choices 41% Food choice limited by caregiver 76% Portion control 35% Meals prepared by caregiver 100%
Describe the food security measures you use	Locks on cabinets / refrigerators 29% Barriers/alarms/cameras 23.5% Constant supervision / line of sight 47% No reported food security measures 35%
If you had to characterize what is most successful in managing the hunger, what would you say?	“We made her world littler [sic], which is sad” “Recognizing the price you pay for going off-schedule” “Routine is the only thing that saves us” “If the opportunity [to get food] isn’t there, she is happier” “100% the locking up. If I forget, she reminds me to lock up. She wants me to lock up. She doesn’t want to have the opportunity to overeat”
What is the biggest challenge in managing PWS?	Behavior/Mental Health management 41% Food management/security 29% “The hard part is worrying about food. It is sickening. It is tiring. I hate it. I hate everything about food” The biggest challenge is “behavior and anxiety, transitioning and managing unmet expectations” “She is not compatible with the world. She is really smart, but she can’t control the food behavior so that takes away all opportunity” “I am tired of living a separate life from my husband. One of us is always on duty and it’s like we live two separate lives”

Limiting social/community engagement where food is present was also frequently reported by respondents. Forty-seven percent (n = 8) of the respondents stated that restaurants and parties with food cause significant distress to the person with PWS, and caregivers reported avoiding these situations to prevent challenging behaviors. Caregivers also frequently report attending social engagements separately as a family, with one caregiver staying home with the person with PWS while the other attends the event or driving separately so one parent can leave with the person with PWS if food behaviors become unmanageable.

A majority of respondents (64.7%, n = 11) implement food security measures in the home, such as locks on refrigerators, pantries, and trash containers, locks on bedroom doors, security cameras and alarms, and physical barriers to the kitchen. Forty-seven percent (n = 8) of respondents report using ‘line of sight’ supervision in which the person with PWS is always within eyesight of the caregiver. Food security measures also extend to outside settings for the majority of participants who routinely spend time in programs and activities outside the home (day programs, camp, employment). Many caregivers themselves report eventually adopting a lifestyle with routine and sameness governing the course of all actions and decisions for the family, to ‘keep the peace’. When considering the possibility of new treatments to manage PWS-associated hyperphagia, most of these caregivers (64.7%, n = 11) indicated that they would be interested in such a treatment if it became available, despite the low HQ-CT score they reported for the individual in their care. The possibility of greater independence for the person with PWS as well as the potential for reduced intensity of caring for the person were the most common reasons for interest in potential hyperphagia therapies.

When asked how hyperphagic behaviors had changed over time for the adult with PWS in their care, most caregivers (53%, n = 9) reported that the food-related behaviors of the person with PWS had stayed about the same, while 12% (n = 2) reported an increase in behaviors and 35% (n = 6) reported decreasing food related behaviors. Of those reporting reduced food related behaviors over time, caregivers cited changes in lifestyle including fewer activities outside the home, school ending, and siblings moving out of the home as contributing to more stability in the management of PWS-associated food behaviors. None of the respondents reported significant lessening of hyperphagia over time in the adult under their care.

## Discussion

Hyperphagia is a hallmark symptom of PWS. The inability to control food intake has a tremendous impact on the ability of the person with PWS to achieve independence and pursue their goals^13^ and contributes significantly to caregiver burden^8^. Thus, it is not surprising that developing new treatments for hyperphagia is the primary treatment preference of caregivers^14^ and individuals with PWS^13^ alike.

The primary clinical outcome assessment for hyperphagia-associated behaviors in PWS is the HQ-CT, a caregiver-reported assessment of hyperphagic behaviors, which has been shown to be sensitive to change in the setting of clinical trials for drugs targeting hyperphagia in the PWS population^15–17^. However, this instrument has not been fully characterized in a large PWS population, nor in typically developing individuals. The present study included a large sample size of typically developing individuals aged 5–26 years, as well as more than 450 individuals with PWS, age 5 and above. As expected, typically developing individuals have significantly lower hyperphagic behaviors as assessed by HQ-CT total score compared to those with PWS, across all ages. Further, typically developing individuals show a decrease in food related behaviors with age, likely reflecting normal maturation, with younger participants more prone to tantrums or persistence in requesting certain foods. In contrast, HQ-CT scores in those with PWS was higher in older individuals, consistent with the natural history of the disorder, where infants show little interest in food, but food interest increases over time, advancing to hyperphagia in late childhood, which persists into adulthood.

In the individuals with PWS studied here, we observe a wide range of HQ-CT scores, with an overall average score of 10.3 out of a possible 36, and a standard deviation of 7.9. The relatively modest average score suggests that not all hyperphagic behaviors assessed on the HQ-CT are observed in all individuals over the two-week recall period. This may be in part because educational efforts for families of those with PWS have focused on implementing food security measures as a means to reduce the prevalence of obesity in this population. Such environmental controls, for example locking up cabinets and refrigerators, may reduce opportunities to access food and potentially result in lower HQ-CT scores. Concurrent assessment of the food security measures implemented by families may shed light on the relationship between HQ-CT scores and environmental control of food access, providing additional context for understanding hyperphagia in PWS.

Interestingly, in both PWS and typically developing individuals, there was a significant difference in HQ-CT scores by weight category, with higher scores in individuals with increased BMI. This finding may reflect the ability of the HQ-CT to capture food behaviors that drive weight gain across populations. However, further research is needed to evaluate the utility of the HQ-CT in non-PWS populations.

A second aspect of this study focused on a deeper exploration of a small, but potentially informative subset of caregivers who report unexpectedly low HQ-CT scores for the person with PWS in their care. It is well known that individuals with PWS display a range of severity with respect to PWS-associated behaviors. The goal of the interviews was to ascertain whether the caregiver felt that hyperphagia was present in their loved one with PWS, but not captured by the HQ-CT instrument, or if the individual with PWS did not exhibit typical hyperphagic behaviors.

Several notable themes were identified during the semi-structured interviews. These caregivers routinely employ a number of strategies to mitigate the food-related behaviors of the person with PWS, including having the caregiver prepare or supervise all meals and snacks, adhering to strict food routines, applying intense diligence regarding food security and supervision, and limiting access to the outside world. For these families, rigid routines and food controls had become a way of life, and caregivers were often habituated to this lifestyle. Most families reported that change in routine happens rarely or never, and caregivers cited concern about problematic food behaviors as the underlying reason to maintain these strict control measures. In such a setting, some of the hyperphagic behaviors assessed in the HQ-CT, (e.g., waking a night to access food, taking food from the trash, stealing food) may not be present because the opportunities to access food are severely limited. Thus, these HQ-CT scores may not reflect what would be seen in a typical, non-food secure environment, rather they likely reflect the considerable effort on the part of caregivers to strictly control the food environment and minimize hyperphagia-related behaviors. Additional studies are needed to determine if these findings are generalizable to the larger PWS population, and to better understand the relationship between food security and hyperphagia assessment in PWS.

It is important to note that despite the food control measures that were used by caregivers, the majority of adults with PWS in this subsample fell into either the overweight (17%) or obese (47%) BMI categories, suggesting difficulty in maintaining healthy weight. Respondents report struggling to control the weight of the person with PWS, but some may have become accepting of overweight or obesity as part of the syndrome. Indeed, in some instances (2 of 17 respondents), the person with PWS is allowed unrestricted access to food. As one respondent commented, the “respite care worker gives her anything she wants. They do not follow her guidelines for food.” In this setting, food behaviors as assessed by the HQ-CT may not be observed because the person with PWS has no food restrictions.

A PWS nutritional “Phase 4” has been hypothesized, where some adults with PWS may no longer experience insatiable appetite^5^. Although our analysis is limited by the cross-sectional nature of the current study and the limited subset of families participating in the interviews, we did not find evidence of Phase 4 in this sample of 459 individuals with PWS, with 84 individuals over the age of 25. Some older individuals who were maintained in a strict food routine appeared to show lower intensity of food seeking behaviors as assessed by the HQ-CT questionnaire, but caregiver interviews suggest that this may not be due to change in hyperphagia per se, but rather achieved by intense supervision, restriction and routine. The limitations in independence and community integration that accompanies such a restricted lifestyle was cited by several caregivers of low HQ-CT scoring-individuals as driving their interest in potential treatments that are being developed for hyperphagia. Thus, even those individuals with PWS with low scores on the HQ-CT could potentially benefit from treatment.

There were a few limitations to this study. First, all the data collected are subject to self-report bias, as the information is reported by the caregivers of individuals with PWS and typically developing individuals. In addition, the caregivers of typically developing children/young adults were asked to self-report the weight category of their child rather than providing height and weight for BMI calculation, which could affect the accuracy of this variable. The study was also limited by the cross-sectional nature of the assessment and the lack of information about food security measures for those with PWS. Ongoing, longitudinal data collection in the PATH for PWS natural history study may be useful in this respect and may allow additional insights into the interplay of PWS-associated behaviors and the environment, as well as the relationship between HQ-CT scores and changes in BMI over time.

## Conclusions

Overall, the HQ-CT captures significant differences in hyperphagia-associated behaviors between typically developing individuals and individuals with PWS. Additional consideration and assessment of environmental controls (e.g., adherence to strict food routine and/or security measures) may be important in fully understanding the degree of hyperphagia in individuals with PWS.

### Supplementary Information

Supplementary Information.

## Data Availability

The data presented in this study are available from the corresponding author on reasonable request.

## Abbreviations

ANOVA: Analysis of variance
BMI: Body mass index
HQ-CT: Hyperphagia questionnaire for clinical trials
IRB: Institutional review board
PWS: Prader-Willi syndrome
