# Supplementary material for: Analysis of Hyperphagia Questionnaire for Clinical Trials (HQ-CT) scores in typically developing individuals and those with Prader-Willi syndrome
Source: Sci Rep. 2023 Nov 23;13:20573. doi: 10.1038/s41598-023-48024-5 (PMC10667498; doi:10.1038/s41598-023-48024-5)
Supplement: Supplementary file 1 — Supplementary Information. [file 41598_2023_48024_MOESM1_ESM.pdf]

Additional File 1

Analysis of Hyperphagia Questionnaire for Clinical Trials (HQ-CT) Scores in Typically Developing Individuals and those with Prader-Willi Syndrome

Lisa Matesevac, Caroline Vrana-Diaz, Jessica E. Bohonowych, Lauren Schwartz, and Theresa V. Strong

Supplemental Table 1: **HQ-CT scores, Question by Question, for Participants with PWS and Typical Individuals**

HQ-CT question	PWS Participants (N=459) (mean ± SD)	Typical Participants (N=614) (mean ± SD)
**1.** During the past 2 weeks, how upset did the person generally become when denied a desired food?	1.47 ± 1.13	0.90 ± 0.82
**2.** During the past 2 weeks, how often did the person try to bargain or manipulate to get more food at meals?	1.34 ± 1.26	0.42 ± 0.74
**3.** During the past 2 weeks, how often did the person forage through trash for food?	0.46 ± 1.07	0.03 ± 0.22
**4.** During the past 2 weeks, how often did the person get up at night to food seek?	0.56 ± 1.25	0.13 ± 0.51
**5.** During the past 2 weeks, how persistent was the person in asking or looking for food after being told "no" or "no more"?	0.99 ± 1.09	0.57 ± 0.75
**6.** During the past 2 weeks, outside of normal meal times, how much time did the person generally spend asking or talking about food?	1.57 ± 1.33	0.49 ± 0.70
**7.** During the past 2 weeks, how often did the person try to sneak or steal food (that you are aware of)?	1.455 ± 1.56	0.55 ± 0.98
**8.** During the past 2 weeks, when others tried to stop the person from asking about food, how distressed did he or she general appear?	1.16 ± 1.09	0.30 ± 0.57
**9.** During the past 2 weeks, how often did food-related behavior interfere with the person's normal daily activities, such as self-care, recreation, school, or work?	1.12 ± 1.21	0.06 ± 0.25

Supplemental Table 2: **Significant Results by HQ-CT Individual Questions in Typically Developing Individuals**

Hyperphagia Questionnaire for Clinical Trials (HQ-CT)			
**HQ-CT Individual Question**	**Age Category**	**PWS Living Status**	**PWS Relationship Status**
**1.** During the past 2 weeks, how upset did the person generally become when denied a desired food?	5-11 y.o.: 1.07<br>12-17 y.o: 0.40<br>18+ y.o.: 0.21<br>5-11>12-17, p<0.0001<br>5-11>18+, p<0.0001	Living with someone with PWS: 0.78<br>Not living with someone with PWS: 0.94<br>t=2.14, p=0.033	
**2.** During the past 2 weeks, how often did the person try to bargain or manipulate to get more food at meals?	5-11 y.o.: 0.49<br>12-17 y.o: 0.21<br>18+ y.o.: 0.03<br>5-11>12-17, p=0.0009<br>5-11>18+, p=0.0031		
**3.** During the past 2 weeks, how often did the person forage through trash for food?			
**4.** During the past 2 weeks, how often did the person get up at night to food seek?	5-11 y.o.: 0.089<br>12-17 y.o: 0.26<br>18+ y.o.: 0.24<br>5-11<12-17, p=0.0038		
**5.** During the past 2 weeks, how persistent was the person in asking or looking for food after being told “no” or “no more”?	5-11 y.o.: 0.64<br>12-17 y.o: 0.37<br>18+ y.o.: 0.069<br>5-11>12-17, p=0.0015<br>5-11>18+, p=0.0002		
**6.** During the past 2 weeks, outside of normal meal times, how much time did the person generally spend asking or talking about food?		Living with someone with PWS: 0.33<br>Not living with someone with PWS: 0.55<br>t=3.44, p=0.0006	Sibling: 0.33<br>Cousin: 0.42<br>Other relation: 0.25<br>Not related to someone with PWS: 0.57<br>Sibling<Not related, p=0.0013
**7.** During the past 2 weeks, how often did the person try to sneak or steal food (that you are aware of)?			
**8.** During the past 2 weeks, when others tried to stop the person from asking about food, how distressed did he or she general appear?	5-11 y.o.: 0.35<br>12-17 y.o: 0.13<br>18+ y.o.: 0<br>5-11>12-17, p=0.0006<br>5-11>18+, p=0.0032		
**9.** During the past 2 weeks, how often did food-related behavior interfere with the person’s normal daily activities, such as self-care, recreation, school, or work?			
